# Computation in Psychotherapy, or How Computational Psychiatry Can Aid Learning-Based Psychological Therapies

**DOI:** 10.1162/CPSY_a_00014

**Published:** 2018-02-01

**Authors:** Michael Moutoussis, Nitzan Shahar, Tobias U. Hauser, Raymond J. Dolan

**Affiliations:** 1 Wellcome Trust Centre for Neuroimaging, University College London, London, UK; 2 Max Planck UCL Centre for Computational Psychiatry and Ageing Research, London, UK

**Keywords:** computational psychiatry, belief updating, Bayesian inference, cognitive-behavioral therapy, mentalization-based therapy, near-miss disaster, avoidance, therapy failure, reinforcement learning, exposure-with-response-prevention

## Abstract

Learning-based therapies, such as cognitive-behavioral therapy, are used worldwide, and their efficacy is endorsed by health and research funding agencies. However, the mechanisms behind both their strengths and their weaknesses are inadequately understood. Here we describe how advances in computational modeling may help formalize and test hypotheses regarding how patients make inferences, which are core postulates of these therapies. Specifically, we highlight the relevance of computations with regard to the development, maintenance, and therapeutic change in psychiatric disorders. A Bayesian approach helps delineate which apparent inferential biases and aberrant beliefs are in fact near-normative, given patients’ current concerns, and which are not. As examples, we formalize three hypotheses. First, high-level dysfunctional beliefs should be treated as beliefs over models of the world. There is a need to test how, and whether, people apply these high-level beliefs to guide the formation of lower level beliefs important for real-life decision making, conditional on their experiences. Second, during the genesis of a disorder, maladaptive beliefs grow because more benign alternative schemas are discounted during belief updating. Third, we propose that when patients learn within therapy but fail to benefit in real life, this can be accounted for by a mechanism that we term overaccommodation, similar to that used to explain fear reinstatement. Beyond these specifics, an ambitious collaborative research program between computational psychiatry researchers, therapists, and experts-by-experience needs to form testable predictions out of factors claimed to be important for therapy.

## INTRODUCTION

Many articles on psychiatric research bemoan the relative shortcomings of psychiatric treatments. Others lament a lack of understanding of the biopsychological processes involved in disorders. We advocate a complementary, positive motive for research. The new field of computational psychiatry studies the computations the brain performs to fulfill human needs in health and during disorder. This research effort can benefit by building on recent advances in mental health treatments. The advances we consider here have been achieved by therapies that focus on learning and inference, therapies with which computational psychiatry has barely engaged. Alternatively, by working with clinicians, computational psychiatry researchers can help answer questions that are clinically important for therapy. In this article, we highlight major research opportunities that stem from closer engagement with psychological therapies.

Learning is necessary for psychotherapy,^1^ which renders important a computational understanding of learning and inference^2^ in therapy. Here we mainly focus on cognitive-behavioral therapy (CBT), which we introduce subsequently. CBT is popular with providers and the public, as its theory is accessible and it is relatively brief, inexpensive, and effective (A. T. Beck, 2005; J. S. Beck, 2011; Lewis & Lewis, 2016). CBT puts general learning and inference at the center of therapy. Other therapies, especially mentalization-based therapy (MBT; Allen, Fonagy, & Bateman, 2008), excel in the special case of interpersonal inference. CBT and MBT are collaborative therapies that teach key aspects of theory to patients, who then use them in the service of change. In this article, we aim to increase computational understanding of these key aspects of theory that are directly used in therapy.

We consider three areas where computational psychiatry can aid learning-based psychotherapies. The first area concerns distortions of inference hypothesized to *maintain* mental disorders in currently unwell people. We prioritize maintenance because correcting such distortions is at the center of psychotherapy interventions. These distortions include the influence of cognitive biases, taken to be distortions of inference, on everyday decision making. We discuss (a) cognitive biases caused by the influence of emotion and (b) inferential distortions caused by behavior itself. Here we focus on avoidance, a behavior that is maladaptive in many disorders (Harvey, Watkins, Mansell, & Shafran, 2004). The second area is *vulnerability*, or predisposing factors for mental disorders. This is important for understanding “what went wrong when” and hence how to devise preventative measures at both the individual and societal levels. The framework of computational psychiatry helps formalize and test existing, semiquantitative therapy hypotheses, especially distorted inferences such as attributional biases, that confer vulnerability. The concluding area concerns shortcomings of psychotherapy. We explain how computational psychiatry could help address these.

## FACTORS MAINTAINING PSYCHOPATHOLOGY

### How Biased Inference Leads to Psychiatric Distress According to Therapy Theories

**Figure 1.**
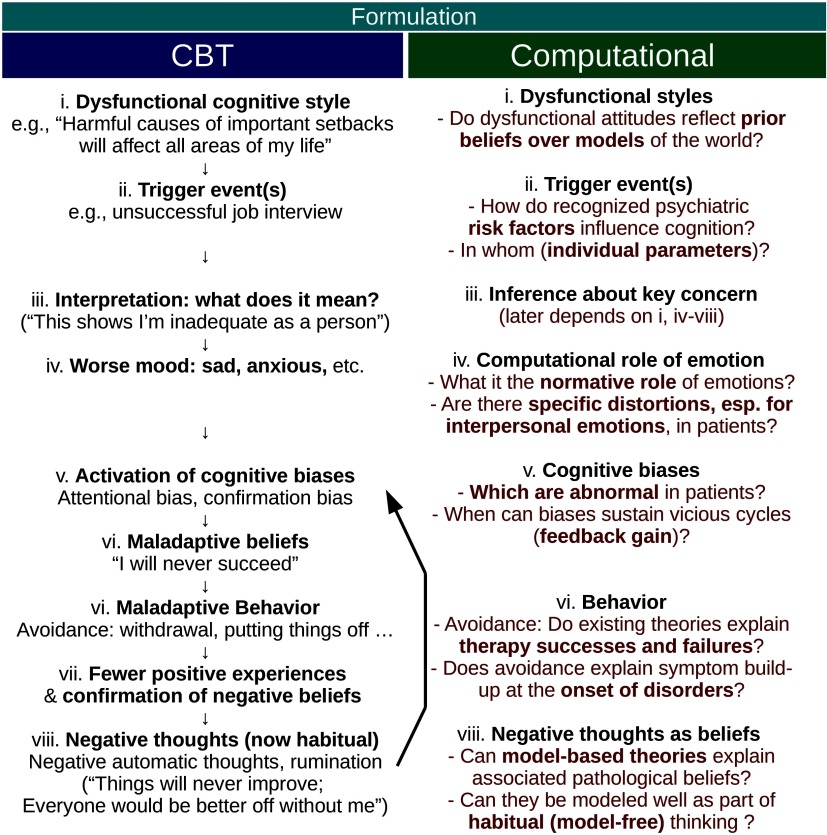
**Correspondence between CBT and computational conceptualizations of a paradigmatic case of depression (Louie in text).** Left: CBT diagram as used in textbooks, in self-help books, and in working with patients with depression. Several aspects of it, such as the postulated vicious cycle including the bold arrow, are still inadequately validated. Right: the postulated CBT process is related to the language of computational psychiatry. Key questions for research are posed in terms of probabilistic reasoning, computational models, and parameters characterizing individuals.

Here we first aim to demonstrate how CBT explains psychiatric disorders, using depression as an example (Figure 1A). In general, such explanations are both part of the theory underpinning CBT and also used in treatment to educate patients, enabling them to collaborate in clinical interventions like the one described in the section “Avoidance Distorts Inference by Impairing Disconfirmation of Maladaptive Beliefs.” The theory postulates that perceived events are in themselves insufficient to induce psychiatric distress (J. S. Beck, 2011). Instead, it is the interpretation of events, or the “meaning” inferred from them, that induces maladaptive behavior and suffering. Suppose a person vulnerable to depression, Louie,^3^ has the following belief: “A big setback means that powerful factors are at play which will harm all areas of my life.” This type of cognitive bias is called a *global attribution* (Alloy, Abramson, Metalsky, & Hartlage, 1988). If Louie experiences a failure in a certain event (e.g., not succeeding in a job interview), a global attribution style might lead him to infer that he is “inadequate as a person” and that future failure is inevitable. Such beliefs may not only induce emotional and somatic symptoms (sadness, loss of energy) but also lead to decisions such as “I will stop trying to avoid further disappointment.” This is maladaptive behavior, as a vicious feedback cycle between avoidance, negative emotion, and inference can then be set up, especially in the presence of cognitive biases. The cycle exacerbates distress (sadness, loss of energy), external problems (no job), and beliefs (self-fulfilling “I will fail”). CBT thus explains depression, both theoretically and clinically, as a cascade of stages, invoking (a) predisposing beliefs (“setbacks mean …”); (b) a trigger event (unsuccessful job interview); (c) maladaptive inferences (“I will never succeed,” “I am inadequate”); and (d) maintained cycles of emotion, maladaptive behavior, and beliefs. CBT accounts of this ilk have been developed for most common mental disorders and are actively used with patients as a basis of understanding psychological distress (Blenkiron, 2010).

**Box 1.** Therapy concepts relevant to inference (alphabetical order).

• **Attribution:** Inference that a particular cause was responsible for an observed event.
• **Attributional bias:** A cognitive bias (see below) affecting attribution (above).
• **Avoidance:** Emission or withholding of activity so as not to enter a feared external situation, as would be ordinarily expected. For example, ‘School avoidance’ = not going to school (Lovibond et al., 2009)
• **Catastrophizing:** A cognitive bias, postulating unwarranted inference of unaffordable and/or severe and irreversible loss.
• **Cognitive (or reasoning) bias:** Propensity to draw inferences unwarranted by the totality of information available to a healthy person in the particular context. Many use it to imply that no qualitative information-processing or organic deficit is present. Hails from learn ing theory and the psychosocial therapies, in opposition to the medical model.
• **Cognitive deficit:** Lack of an adaptive information-processing function. Many use it to imply that a reasonably well-defined brain problem (gene, lesion, mis-development, etc.) adversely affects information processing. It hails from neuropsychology / medical model. Computational psychiatry elegantly reconciles ‘bias’ and ‘deficit’.
• **Dysfunctional assumption:** Belief such as ‘unless I always succeed, I’m a loser’, used as a rule guiding behavior (‘... so I must always succeed’). It can lead to unhelpful inference (‘I failed in this, what a loser I am!’) and hence psychopathology.
• **Ex-consequentia emotional reasoning:** Treating the presence of an emotion as evidence for congruent inferences-e.g., ‘I am anxious, therefore there is danger around’. Tradi tionally considered a reasoning error, we argue that it is in the first instance an adaptive process of inference on the basis of interoception—though of course only in the first instance.
• **Exposure:** Entering the feared situation, whether withholding or emitting safety behaviors. See also ‘response prevention’.
• **Mentalizing:** Inference of mental states as causes of the intentional behavior of self and others, relevant to the self.
• **Mentalizing breakdown:** Stereotyped inference about mental states, usually resulting in unwarranted inferences that take place under strong emotions. Closely related to mind- reading (below), but more elaborated, as it’s used in conditions where such biased theory- of-mind is of central importance.
• **Mind-reading:** A cognitive bias, postulating an overconfident inference that others hold negative views about the self. Note that in CBT this term is more narrowly defined than in common parlance.
• **Psychotherapy:** A formal treatment aiming to change mental function and perception by using existing sensory inputs in order to improve mental health. Here we include at least the psychological therapies of all modalities (behavioral, analytic, systemic, etc.) and the rehabilitation therapies.
• **Response-prevention:** Withholding safety behaviors (see below) upon entering the feared situation (what happens in Exposure with Response Prevention, or ERP).
• **Reinstatement:** The re-appearance of an older pattern of behavior in response to a stimulus, well after new behaviors have been successfully learnt, as a result of non- specific factors such as the passage of time or the occurrence of non-specific stress.
• **Schema:** Constellation of properties pertaining to a state, and appropriate actions to be performed in response. A person’s mini-model of an aspect of a situation. Some therapy theorists write about emotion as part of schema, but here we follow the more traditional analysis of emotional feelings as interacting with schemas which include beliefs about self, world and appropriate action.
• **Safety-behavior:** In general, any behavior carried out in order to reduce or avert a serious feared outcome (Salkovskis, 1991). In that sense avoidance is a safety behavior that removes the patient from the anxiogenic situation. However ‘safety behavior’ usually means usually means protective behaviours emitted within the anxiogenic situation.

A similar process of disorder maintenance holds true for interpersonal inference, as portrayed in mentalization-based therapy and its account of emotion. MBT is an effective therapy in reducing suicidal behavior and severe self-harm (Bateman & Fonagy, 2008). Here *mentalizing* means “inferring about intentional mental states of others relevant to the self,” and MBT specifically targets patients’ capacity for such inference. In particular patient groups, commonly those with a history of severe adversity (childhood neglect, physical and sexual abuse in adolescence), current interpersonal difficulties trigger strong negative emotions. MBT describes how, in such patients, emotions guide the formation of beliefs about other people, such as “others hate me.” These lead to a generalized dysfunction of the process for inferring mental states. This frequently results in overcertain, catastrophizing inferences about the self and others. Extreme emotions and destructive behaviors then worsen interpersonal difficulties and foster maintenance of the disorder. This cycle is analogous to that of Figure 1, but specific to interpersonal emotions and beliefs. MBT addresses these generalized problems of inference by facilitating consideration of a broader range of mental state attributions and by training patients to detect inadequate mentalizing (mentalizing breakdowns). This benefits patients (Bateman & Fonagy, 2008) but is often hard to do, as exemplified by the following patient blog post:^4^

I am feeling very paranoid. … I tell myself that it must be true: She truly does hate me. … I am able to Check The Facts: perhaps she [is not answering my e-mail because she] is just super busy. At the same time, being able to rationalise in this way doesn’t take away from the mental chaos evoked in me by her lack of a response.

The dynamics of emotion and how they interact with maladaptive inferences, an area of much discussion among therapists, thus need to be much better understood. We discuss them in the following pages, especially in the section “How Emotion Helps Construct Meaning.”

In this article, we examine key mechanistic claims that therapy theories make and therapists themselves use with patients because, despite the considerable efficacy of learning-based therapies, the mechanisms through which they achieve their effects are uncertain. First, we examine whether avoidance can truly maintain and exacerbate distorted inferences, maintaining psychopathology. Second, we discuss whether cognitive biases and negative emotions form clinically important vicious cycles. Third, we consider the specific role that emotion may play in distorted inference. In each case, we introduce the vocabulary and formalism of computational psychiatry, enabling us to identify important limitations of current theories and formulate new hypotheses.

### Etiological Role of Avoidance

#### Avoidance distorts inference by impairing disconfirmation of maladaptive beliefs

In CBT, a key factor that can bias inference toward maintaining maladaptive beliefs is behavior itself (Lovibond, Mitchell, Minard, Brady, & Menzies, 2009; Salkovskis, 1991). If a situation is believed to be risky, and the risk is mitigated through avoidance or safety behaviors, it becomes difficult to find evidence disconfirming the belief about this situation being risky, even if it is (now) safe. In social anxiety, for example, avoiding social exposure prevents disconfirmation of the belief that otherwise one would be ridiculed (Moscovitch, 2009).

One way CBT tries to induce change in maladaptive inferences is by encouraging what it terms *behavioral experiments*.^5^ In a behavioral experiment, the patient is usually asked to monitor and rate a specific belief (e.g., 0%–100%) and *perform behaviors through which belief-relevant evidence is elicited*. Therapists will usually encourage a patient to note a discrepancy between expectations based on the initial (prior) belief and the current evidence gathered in the behavioral experiment, hoping thereby to shift the maladaptive belief. For example, a patient whom we will call Susie^6^ suffered from social anxiety, leading to a reluctance to enter public places, as she was afraid that her looks would be mocked. She specifically avoided entering coffee shops, expressing the belief that “everyone entering a coffee shop is very well dressed.” Feeling she could “never dress right,” she avoided these public places. In therapy, she was encouraged to consider an experiment to enable her to check on her beliefs. Determined to deal with her avoidance, she decided to stand outside a coffee shop for 1 hour, protected from others’ view, and record the appearance of people entering. Needless to say, most people she observed entering the coffee shop were casually dressed (and suffered no ill effects). According to her own account, noting this fact alone gave her enough courage to start entering coffee shops. Let us now rephrase the patient’s account in computational terms. The new inference “being casually dressed in coffee shop → no ridicule” is, from a computational perspective, a lowered estimate of the probability to transition from the state “casually dressed in coffee shop” to the state “ridiculed.” This is useful for model-based decision making. As a consequence of belief change, this patient could update how risky her own relevant actions would be, thus updating so-called action values. Here the negative value of “I enter the coffee shop” substantially improved (see Box 2 for terms). In computational parlance, learning is updating beliefs about one’s models of the world, so Susie has learned something very useful.

In contrast to the behavioral experiments, exposure-based techniques help without explicitly addressing model-based accounts of the world. They update effective^7^ beliefs without overtly discussing these beliefs. A classic example here is exposure-with-response-prevention (ERP), which is the first-line treatment for adult obsessive-compulsive disorder (OCD) in England.^8^ Here patients agree to gradually enter a feared situation (*exposure*) while withholding any behaviors they ordinarily perform there (*response prevention*) to avoid harm.^9^ Related exposure approaches are successful in many disorders, including depression (Veale, 2008). These therapeutic advances raise important computational questions regarding *how* exactly they work and under what conditions they may *fail* to help. We now turn to the how, addressing the computations involved in belief disconfirmation, and examine the question of therapy failure in the section “Therapy Failures and the Possible Role of Overaccommodation.”

#### Avoidance can slow down disconfirmation of beliefs but cannot increase them

Here we first show how models of inference explain how avoidance dramatically dampens cessation of safety behaviors and associated belief disconfirmation. Intuitively, if Susie were anxious about coffee shops but never explicitly thought about them or approached them, her anxiety would not be extinguished. However, later on in this section, we will show that avoidance theory is poor in explaining how maladaptive beliefs grow in the first place, as disorders develop. In this section, we use *belief* broadly, to include that the individual considers a particular state highly aversive even if a bad outcome is not delivered at the state in question (Sutton & Barto, 1998). Thus we subsume in the term *belief* accessible, explicit beliefs; inaccessible effective beliefs; and formal beliefs. Beliefs about the future are often implicit, for example, if the anxious Susie has little explicit idea about coffee shops. A therapy different to the earlier described one might then expose her to stimuli gradually approximating the feared one until she learned, by simple association, that *not* avoiding coffee shops incurs *no* costs.

**Box 2. Computational concepts (derivative concepts appear lower).**

- **State space:** Set of mutually exclusive sets of features that define the structure of the situation at hand—including the allowable transitions—and so provide agents with a complete repertoire on the basis of which to form beliefs about the situation. The state space {0,1} has states with no further structure while the space {she loves me, she loves me not} has states that may be described by different, complex models.
- **Action:** Choice that an agent makes which impacts on their trajectory through the states visited and returns obtained, and which includes physical and/or mental / computational actions.
- **Value:** a quantitative property of a state (state value) or an action (action value) that determines how likely it is that, given the choice between occupying this state (or action) and some alternative, a decision-maker will choose to visit the one in question. Higher- value states (or actions) are chosen more often but their phenomenal and neural correlates may vary.
- **Belief:** Formally, probability distribution over a set of states of the world, including the self. See, however, ‘effective belief’ and section Aetiological role of avoidance.
- **Effective belief:** Belief that would lead the decision-maker to behave (e.g., decide) as they do, as opposed to an ‘explicitly represented belief’ where the representation itself is in some sense measurable, for example by self-report. ‘Effective’ thus does not mean ‘useful for achieving goals’.
- **Inference:** The updating of beliefs about the state of the world and the consequences of actions. The ‘state’ may include states of the self and which model of a process may be true.
- **Bayesian inference:** Updating of beliefs qua probabilities on the basis of previously unconsidered evidence (or datum, or observation). Under quite general assumptions it is the optimal way of updating beliefs.
- **Learning:** The updating of one’s model of the world. Which model to have can itself be considered a belief, and therefore learning is just inference about the model of the world. We will mostly use ‘inference’ unless it is important to emphasise that updating the model of the world, as opposed to which state the world is in, is at stake. In the latter case we will follow the conventional distinction above.
- **Likelihood:** The probability that a piece of data will be observed given a set of beliefs (i.e. a model/cause), notated Pr(data — hypothesis). This includes a specification of how the data is produced by underlying causes.
- **Prior belief:** Belief before evidence was taken into account and inference performed.
- **Posterior belief:** Belief after inference was performed, a.k.a. conditioned on the evidence.
- **Model-based, goal-directed or outcome-driven decision-making:** Decision making ex plicitly representing state transitions towards outcomes. In the example of Susie who has social phobia, ‘If I enter the coffee-shop, well-dressed people will be there, they will notice my clothes, and will mock me. Therefore entering the coffee-shop is bad’.
- **Model-free**, cached-value or associatively driven decision-making. If Susie doesn’t think explicitly about the future, she may act upon experience having taught her that ‘Entering coffee-shops brings trouble. That’s all I keep in mind’.

**Figure 2.**
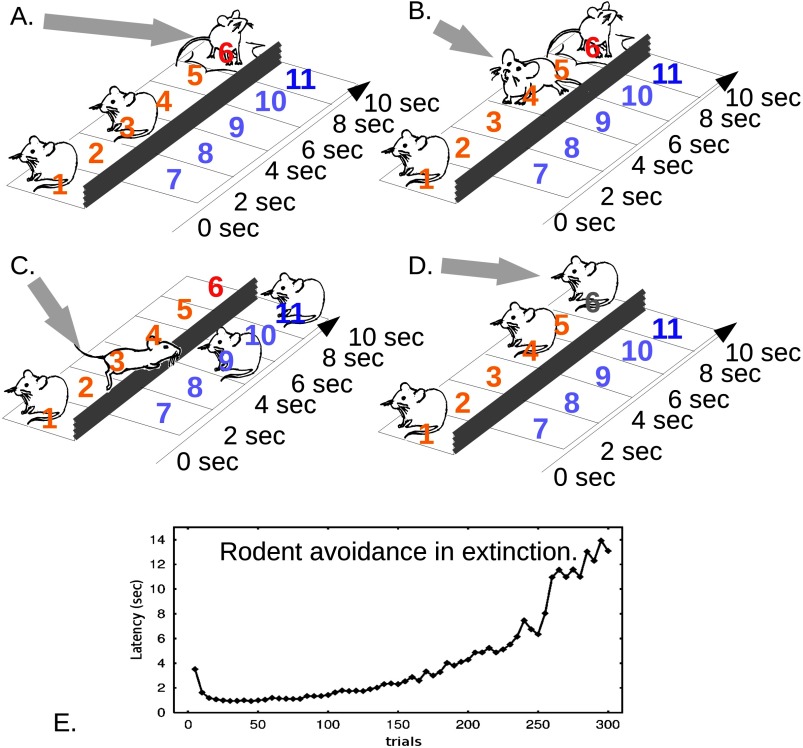
**Avoidance learning and spontaneous extinction.** Each trial starts on spatiotemporal state 1, signaled to the agent by a light. All possible states are enumerated. Time moves the agent up and to the right. There are two spatial states, left and right of the gray barrier. A small cost is needed to perform avoidance, that is, to jump the barrier toward the safe states 7–11. A) Punishment. Before learning avoidance, the agent does nothing, and time alone lands it in State 6, where it receives the adverse outcome: a shock (gray arrow). B) Acquisition of negative state value. Earlier states quickly acquire negative value by association, but avoidance has not yet been learned. C) Avoidance. Jumping is acquired. D) Extinction. The shock at State 6 has been turned off, and avoidance wanes after a number of unshocked trials. E) Acquisition and loss of avoidance in extinction. Before Time 0, the avoidance action is not available, and shocks are received ˜10 s after the light (as if States 7–11 are unavailable in A–B). At Trial 0, the avoidance response becomes available (7–11 in A–D), *and the shocks are switched off*. Rodents show vigorous reduction in the latency of avoidance during Trials 1–20; that is, they behave as in C despite State 6 now being innocuous. Avoidance is maintained for many trials in extinction (i.e., without any adverse outcomes) but eventually decays. After 270 trials, the rodents remain for longer than the previously shocked latency on the previously shocked compartment. Adapted with permission from “A Temporal Difference Account of Avoidance Learning,” by M. Moutoussis, R. P. Bentall, J. Williams, and P. Dayan, 2008, *Network*, *19*, p. 140.

We now turn to a computational description to provide more insight into how avoidance slows down belief disconfirmation. Active avoidance is shown in Figures 2 (experiment) and 3 (modeling). The agent goes through a sequence of states as time passes, finally arriving at a harmful or safe outcome. The agent may, but does not need to, have a representation of the transitions “I think State 1 will lead to State 2, eventually to State 5, then to harm at State 6.” Purely experiencing “1, 2, … , 5, harm” repeatedly can propagate aversive value down to States 2 and 1 (see Box 2, model-free decision-making). Taking vigorous avoidance action from State 1 to a safe state associatively renders State 1 less aversive. Thus freely performed avoidance can take place in the absence of significant negative emotion. Now suppose State 6 no longer incurs harm. Vigorous avoidance at State 1 means that State 2 is rarely experienced, so State 2 and subsequent states remain negatively charged. The effective belief “1 → 2 … → harm,” even if not explicitly represented but only reflected in the aversive values of States 2 onward, will then change slowly. Thus avoidance blocks learning.

**Figure 3.**
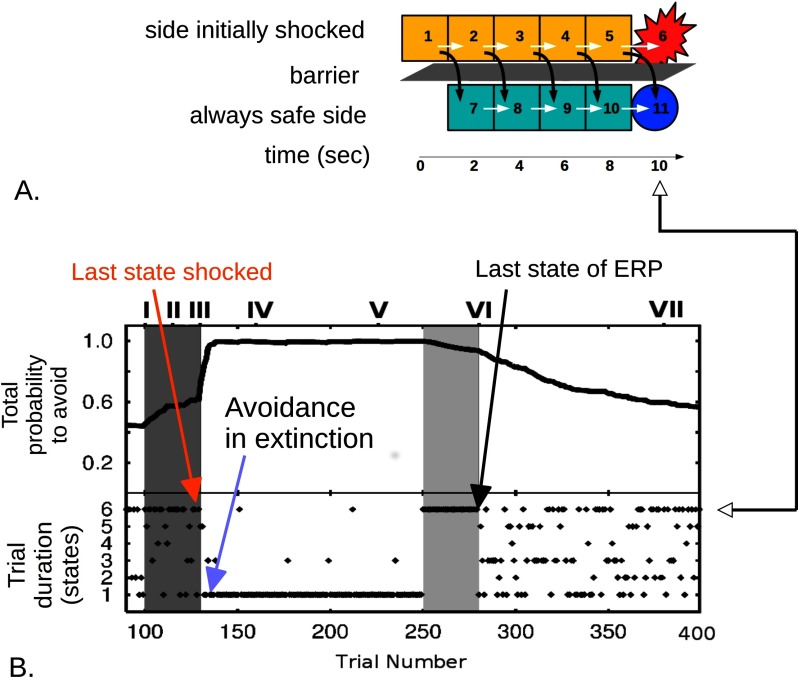
**Modeling avoidance learning, exposure-with-response-prevention, and spontaneous extinction.** A) State space labeled as in Figure 2. The white arrows are the action “stay,” while the black curved arrows indicate “jump to safe state.” B) Simulation of associative, model-free learning: I–III (dark gray), exposure to harm while avoidance is inhibited by the experimenter; III–IV, rapid establishment of effective avoidance when it is no longer inhibited, in extinction (this is entirely analogous to Trials 0–20 in Figure 2E); IV–V, as in human psychopathology, here vigorous avoidance dramatically impairs its own extinction (this mimics behavior around Trials 20–100 in Figure 2E); light gray (to VI), therapy-like ERP, where the mouse is prevented from carrying out avoidance behavior and thus learns that State 6 is no longer dangerous; VI–VII, further gradual extinction of avoidance. The reader is referred to Moutoussis et al. (2008) for further technical details, including the relevant neuropharmacology. Adapted with permission from “A Temporal Difference Account of Avoidance Learning,” by M. Moutoussis, R. P. Bentall, J. Williams, and P. Dayan, 2008, *Network*, *19*, pp. 144, 150.

This impairment of learning through avoidance, and how ERP overcomes it, are detailed in Figure 3B. Here strong aversive values were first established by restricting the agent from performing efficient avoidance during early, shocked trials. When this restriction was lifted, the agent quickly learned avoidance straight from State 1, ameliorating its value. However, the aversive value of States 2 onward changed very slowly, to the extent that States 2–6 were experienced through rare mistakes and, in not leading to harm, became slightly less aversive (Epochs III–V). Efficient avoidance dominated. Subsequently (light gray to VI), ERP was modeled. The avoidance action was made totally unavailable, so that the agent had to follow “1 → 2 … → 6,” but the shock in the previously punished State 6 was omitted. Modeling showed that exposure and response prevention did not have to fully reverse the aversive values, or implicit beliefs, of States 1–6; it just had to reduce them enough to “unstick” decision making so that the agent could learn from further experience (Moutoussis et al., 2008). Returning to Susie, as long as she now went to coffee shops, their negative value and her anxiety would gradually subside.

We now turn to why avoidance and analogous safety behaviors are an inadequate explanation for stable belief maintenance, let alone the growth of such beliefs during development of the disorder, despite being able to dramatically slow down disconfirmation of negative effective beliefs. Agents do not always perform perfect avoidance. The rodents in Figure 2E will occasionally jump to safety a little later. When they do so, this later state—for example, State 3, 4 s into the trial—will go unpunished. Learning will blunt its aversive value and so it will be visited more often. Gradually, avoidance will happen later and later and will eventually cease spontaneously. This is exactly what the model in Figure 3 also predicts (Moutoussis et al., 2008). The analogue of this does not seem to happen in psychiatric disorders. In recognition of this problem of avoidance theories, several hypotheses have been put forward (Gray, 1987; Rachman, 1984; Salkovskis, 1991). These postulate additional factors, for example, within-situation safety behaviors, and the reader is referred to standard CBT texts for more detail (Harvey et al., 2004). However, these theories cannot resolve the issue because they do not represent violations to Bayesian updating, plus some rather general assumptions appropriate to the situation of avoiding harm, as we will explain shortly.

The biased information gathering and biased starting beliefs implied by avoidance theories are inadequate to explain increasing anxiety behaviors in general, not just in the paradigm described so far. A formal exposition is provided in Box 3 for the interested reader, but the syllogism is as follows:

In the period when avoidance (or safety behaviors) builds up, it is not universally performed, and there will be many occasions when no actions or percepts signaling safety occur.
2. On those occasions, no catastrophe materializes, as the situation is in fact safe.
3. Then some learning occurs that the presumed unsafe situation is in fact safer than was thought.
4. Therefore avoidance driven by this belief must decrease. In addition, if the situation is in fact safe, the occasions when successful avoidance is performed can at worst provide no evidence either way. Therefore overall avoidance/safety behavior cannot increase but must in fact decrease, if slowly.

However, Bayesian reasoning (see Box 3) indicates that Step 3 will only happen if the agent treats consistently the possibility that a benign cause of his or her experience operated in the first place, even if this possibility, or prior belief, is small (but nonzero). In the next section, we consider a promising alternative mechanism that does violate this syllogism.

#### Counterfactual thinking about “near-miss disasters” may exacerbate maladaptive beliefs

We suggest that a consideration of counterfactual outcomes (i.e., outcomes that did not materialize), when these are consistent with maladaptive beliefs, may block the update of alternative, more realistic beliefs. Counterfactual thinking can then strengthen maladaptive beliefs and drive an increase of avoidance behaviors in the absence of actual feared outcomes. Modifying beliefs in therapy is a very challenging task for both patients and therapists. Patients report persistence of maladaptive beliefs, even when challenged by strong counterevidence. We suggest that during the development and maintenance of a disorder, this failure in belief update may **Box 3.** Bayesian updating increases belief in benign causes of events ‘in extinction’.

Here, we demonstrate how belief in the presence of benign causes of experience must increase on the occasions where (i) no avoidance / safety behavior is performed in (ii) a situation previously thought to be dangerous, yet (iii) the feared catastrophe does not materialize. Thus, we show how general the conditions are that, under Bayesian updating, render unstable the (maladaptive) belief that harmful rather than benign causes give rise to experience. Let return *r*_*b*_ belong to the set of all benign returns. We divide causes into benign ones, that cannot produce the feared catastrophic outcomes, and harmful ones that can. The prior belief *p*(*C*_*b*_) that benign causes *C*_*b*_ have given rise to a non-catastrophic outcome, forms the basis for updating to the posterior *p*(*C*_*b*_|*r*_*b*_). We use Bayes’ rule to decompose the prior belief that a benign return would materialize in the denominator of the following expression:

$$\begin{array}{lll} p(C_{b}|r_{b}) & = & \frac{p(C_{b},r_{b})}{p(r_{b})}\\ & = & \frac{p(r_{b}|C_{b})p(C_{b})}{p(r_{b}|C_{b})p(C_{b})+p(r_{b}|C_{h})p(C_{h})} \end{array}$$

As benign causes cannot produce the feared outcomes, *p*(*r*_*b*_|*C*_*b*_) = 1. Furthermore, we consider that agents are neither absolutely certain that benign causes obtain (totally naive) nor completely certain that they do not obtain, i.e. 0 < *p*(*C*_*b*_) < 1. This is indeed the case for someone with as yet moderate anxiety. In addition, *P*(*r*_*b*_|*C*_*h*_) < 1 because the harmful cause by definition produces feared outcomes with positive probability. Hence,

$$\begin{array}{lll} p(C_{b}|r_{b}) & = & \frac{p(C_{b})}{p(C_{b})+p(r_{b}|C_{h})(1-p(C_{b}))}\\ & > & p(C_{b}) \end{array}$$ (1)

Equation 1 shows that the dangerous situation must now be regarded as more benign. The conditions for this are that the rules of belief update are not violated and, crucially, that the agent consistently uses their prior belief that benign causes *C*_*b*_ may have operated, however small but non-zero this is.

reflect a dominating impact of the counterfactual, worst-case scenario. This could prevent relatively benign and more realistic beliefs from being reinforced. If events are not attributed to a relatively benign cause, then the worst-case beliefs and the consequential avoidance or safety behaviors will be strengthened instead. We term this rerouting of reinforcement, now directed to maladaptive beliefs, *counterfactual gating*.

We suggest that a computational account of counterfactual reinforcement can be based on *near-miss inference*. Near-miss inference is the strengthening of the (effective) belief that A leads to B, upon observing A in association with an event B’ that resembles B. Near-miss inference was first described in gamblers (Griffiths, 1990). Here losses consequential to outcomes that are conceptually or perceptually close to those signaling wins have been shown to reinforce gambling, despite the gamble having led to a loss. For example, suppose that in a fair dice-rolling game, only the number 6 wins. A near-miss inference would be, upon drawing a 5, “I nearly won; the next draw is likely to be 6.” Our gambler’s effective belief about the world is that some cause is currently present that produces draws around 5, so 6 is likely, whereas actually rolling 6 is independent of having rolled 5. Such losses (near-misses) recruit ventral striatum and dopaminergic midbrain (Chase & Clark, 2010) much like monetary wins, suggesting a candidate neurobiological substrate for appetitive-near-miss inferences.

Near-miss inferences have been described in the development of anxiety conditions, where they apply to forming beliefs about a harmful event occurring (Lovibond, 2001). Here the harmless outcome of an event does not mean that the event was not a “near-miss disaster.” Therefore a seemingly safe event might paradoxically be rendered as evidence that the situation is unsafe; that is, exposure to an event believed to be dangerous but where no harm accrues leads in some individuals to counterfactual reinforcement. For example, if Susie’s friend took her to a coffee shop and she got anxious, she might deduce that “going to the coffee shop almost put me in harm’s way” rather than “the fact that no one mocked me means that it’s safer than I thought.” Even if a person avoids a situation believed to be dangerous, the person’s model of the world may lead him or her to learn counterfactually, for example, on the basis of the person’s own anxiety symptoms. Verbally, this may be expressed as “if I am afraid, it means something really bad nearly happened.”

To understand why anxious people, or gamblers, do not gradually learn that their effective beliefs about models of the world are unwarranted, additional factors must be invoked. This is about longer-term learning rather than immediate inference. At the level of the brain, an overinfluential model of malign causes being at work may block learning about benign causes of events. The simplest mechanism of blocking such learning would take place in the presence of anxiety, wherein patients overly focus cognitive resources on threat, ignoring the scenario whereby near-miss stimuli were produced by relatively benign causes. The effective prior belief over benign causes is discounted, even down to zero, violating the inferential process described earlier (also in Box 3). In the mirror-image gambling situation, to strengthen near-miss-related beliefs, pathological gamblers must block updating (here weakening) the belief that similarity implies correlated occurrence. We would appeal to counterfactual gating of reinforcement to explain these cases.

The neurocomputational substrates of near-miss losses and counterfactual reinforcement of maladaptive beliefs remain unclear (Coricelli, Dolan, & Sirigu, 2007). Future neuroimaging of the neural representations of aversive near-miss and their hypothesized computational roles should help us understand psychopathology. With respect to near-miss losses, we hypothesize that neural activity will correspond to the activity accompanying actual punishments, expressed as aversive prediction errors. This can be tested simply by inverting the valence of outcomes of existing paradigms. For example, a key study used a simplified slot-machine framework to compare neurobiological and behavioral responses to full miss, near-miss win, and occasional win outcomes. Here the participants were pathological and nonpathological gamblers (Chase & Clark, 2010). For the purposes of investigating the influence of near-miss aversive events, the valence of outcomes of this paradigm would be inverted. Near-miss large monetary losses (actually entailing no loss) would be compared with full-miss (clearly safe) events and occasional large losses in healthy and anxiety-prone participants. With respect to counterfactual gating, experiments would contain enough near-miss losses for participants to learn that these are in fact equivalent to full misses. This is the analogue in the aversive domain of learning that rolling a 5 is in fact irrelevant to rolling a 6. However, we predict that individual variability in such experiments, which will prove relevant to psychopathology, will be best elicited by scenarios that depict patients’ concerns. In the case of patients vulnerable to anxiety, we expect the scenarios most likely to be effective to be framed in terms of danger, as we have done in recent work (Nord et al., 2017).

### Postulated Vicious Cycle of Cognitive Bias and Negative Mood

We now turn to the next pathogenic mechanism postulated by CBT, namely, that negative mood and biased attribution of meaning worsen each other by forming a vicious cycle (Figure 1). Several studies have suggested that the mechanistic importance of the cycle is doubtful. Invoking this inference cycle is clinically useful as it makes intuitive sense to patients and motivates working with their inferences, but it may simply be wrong as a theory of disorder maintenance.^10^ Several biases have been thought to contribute to pathology by overemphasizing information around a key concern (Harvey et al., 2004). This includes the *availability heuristic*(bias toward inference supported by easily recalled examples), *confirmation bias* (more weight on evidence supporting currently preferred hypothesis), and others. However, evidence suggests that such biases are not specific to clinical populations. When healthy people are immersed in contexts like those that preoccupy patients, they often display similar biases (Jong, Merckelbach, Bögels, & Kindt, 1998; Pury & Mineka, 1997). In almost every other case examined, exaggerated biases in patients relate to a key area of concern, for example, negative views of self in depression (Harvey et al., 2004). It is uncertain if these biases are in fact aberrant, if they maintain pathology, and if therapy *needs*to address them.

Computational modeling can help test whether the cycle “key-concern activation → belief → biased information sampling → belief maintenance …” is quantitatively important for disorder maintenance. Negative mood is suggested to result from beliefs but also to strengthen the arrows in the cycle. Computational modeling can determine characteristics (e.g., degree of updating of beliefs given sampled information) that can stabilize such cycles (Figure 1). For some, biased information sampling may be appropriate *given* their key concerns, whereas in others, it may be exaggerated and thus drive the cycle. We suggest that for each postulated bias, an information sampling–belief model is set up and the gain of the postulated feedback loop (Figure 1) is examined as a function of the individuals’ model parameters (Hauser et al., 2017), first in silico and then in vivo.

### How Emotion Helps Construct Meaning

Here we aim to translate the core claim of CBT and MBT regarding the crucial role of emotion in the construction of meaning, including pathological meaning, into a computational role for emotions. We formalize human emotions as providing rich prior beliefs about both the state that the self is in and, importantly, an appropriate set of actions. Belief about state-and-actions is then a formalization of a schema, first introduced by Piaget as a set of interlinked representations used both to understand the meaning of and to respond to a situation (Piaget & Cook, 1952). We suggest that emotion provides a “belief” here in the formal sense of informing which schemas are probably useful, while remaining entirely open as to its phenomenological and biological content. Next, we formalize the meaning of an event as the posterior belief over schemas given the event. Thus the role of emotions is to aid the computation of this meaning, that is, posterior belief. The priors informed by emotion would focus further inference, based on additional features of the state of the self and the world, toward the schemas most likely to be useful.

How might this emotion-mediated process help inference? Brains can handle (quickly and precisely) sensory characterization tasks, for example, decoding facial expressions. Characterization includes feeling emotions in response to events. Conversely, we have a very limited capacity to perform explicit probabilistic reasoning, such as Bayesian inference. Emotions may thus be a way to characterize very complex, often social events in terms of small, manageable sets of schemas, which may then be processed more explicitly and in more detail. Suppose that Louie, in our example of depression, goes for a job interview. He faces an interview panel that includes a chair who makes eye contact and looks unsmiling. Louie, like most humans would, has a rapid emotional response to this event. One possible emotion might be to “feel confident”; another might be to feel “intimidated.” Probabilistic reasoning can help determine when this emotional process usefully guides cognition and thus increase its efficiency. Here we provide the conceptual essence of this reasoning, while Box 4 is provided for readers interested in a more formal account. Emotions would be useful if, first, each emotion focuses cognition to a limited, manageable set of schemas. If possible, this would be just the best schema or a very small set. Second, efficiency is subserved by the most appropriate emotion (rather than an unwieldy, complex mixture) being inferred from the event. Here the emotion “confident” would increase belief in the state “the chairman is neutral” and the action “show enthusiasm,” while “intimidated” would increase belief in “aggressive chairman” and “look down.”^11^ These would have very different effects on how the interview goes!

The mentalizing breakdowns of MBT translate neatly to this formulation. In the event of a relationship difficulty, inference of emotion by a patient may result in a high probability of “feeling very upset.” Inference on the basis of “very upset” would highlight unhelpful schemas, including self-harm. Schema-focused CBT has also elaborated upon and applied Piagetian schemas (Giesen-Bloo et al., 2006), and further therapy variants have been developed. Here we make a first suggestion and point out the urgent need to formulate concise models of these clinical accounts of interpersonal emotions that can be tested experimentally.

## VULNERABILITY CONFERRING HIGHER ORDER BELIEFS

Predisposition theories postulate that beliefs formed before the onset of psychiatric disorder form the basis upon which inference about life events precipitates symptoms (J. S. Beck, 2011). They thus explain why the same events do not precipitate disorders in all those so exposed. Researchers have made substantial efforts to prospectively evaluate whether such belief structures *in the absence of disorder* predict its latter occurrence. When it comes to depression, important predisposition theories include the *negative self-belief*(A. T. Beck, 2005; J. S. Beck, 2011) and *hopeless attribution* (Alloy et al., 1988) theories. According to the latter, the inference “I have been made redundant, therefore I will never succeed” becomes understandable if the patient held a *Weltanschauung* (world model) whereby “harmful causes of important setbacks will affect most areas of my life” (Figure 1). Abramson and coworkers called this a *global attributional* style (Alloy, Peterson, Abramson, & Seligman, 1984).

In a recent study, Pearson and coworkers (2015) showed that hopeless attributions (like our example in the section “How Biased Inference Leads to Psychiatric Distress According to

**Box 4.** How emotions may aid inference over schemas.

Here we examine conditions under which emotion simplifies the selection of useful schemas. Let us denote beliefs as *p*, schemas as *S* and emotions as *E*. We formalize the meaning of event *A* as the posterior beliefs about *S* given A, *p*(*S*|*A*). To estimate this, we consider the various emotions *E* that *A* may induce and marginalize over them:

$$\begin{array}{lll} p(S|A) & = & \underset{all\;E}{\sum }p(S,E|A)\\ & = & \underset{all\;E}{\sum }p(S|E,A)p(E|A) \end{array}$$ (2)

where we used the definition of joint probability to write *p*(*S*,*E*|*A*) = *p*(*S*|*E*,*A*)*p*(*E*|*A*). Equation 2 is cumbersome if all possible emotions have to be considered. Emotions become computationally efficient if *p*(*E*|*A*) is large only for a few *E*, reducing the sum to just a few terms. For this to work well, emotions must be rich enough to allow access to all the best schemas through *p*(*S*|*E*,*A*). The Equation 2 simplifies even more if *E* mediates most of the dependence of *S* on *A*, so the *p*(*S*|*E*,*A*) reduces approximately to *p*(*S*|*E*).

p(S|A)∼p(S|E)p(E|A) (3)

When this approximation is good then emotions can increase decision-making efficiency, reflected in the simplicity of Equation 3. Emotions may also be useful if *p*(*S*|*E*,*A*) approximately factorises into a dependence upon emotionally relevant data *A*_*r*_ which give rise to *E*, and emotionally neutral data, *A*_*n*_:

$$\begin{array}{lll} p(S|A) & = & \underset{all\;E}{\sum }p(S|A_{n})p(S|E)p(E|A_{r})\\ & = & p(S|A_{n})\underset{all\;E}{\sum }p(S|E)p(E|A_{r}) \end{array}$$ (4)

Here, only the relatively few schemas compatible with the dominant emotions of the sum term will need further consideration in the light of the emotion-neutral data.Therapy Theories”) predicted future depressed mood. More specifically, once controlling for current mood and other factors, only global attributions predicted future low mood. Global attributions are not just about the cause behind an event but about the way in which this cause is likely to operate in the person’s life. We can conceptualize this as an aspect of model likelihood of how causes operate. If this aspect of *Weltanschauung* is biased, updates of downstream components will be skewed. More generally, if a number of different models are considered by the patient, some with global causes operating and some with specific ones, we can think that the patient is faced with a problem of selecting between them. Attributional style can thus be thought of as a *prior belief distribution privileging some models* over others.

**Figure 4.**
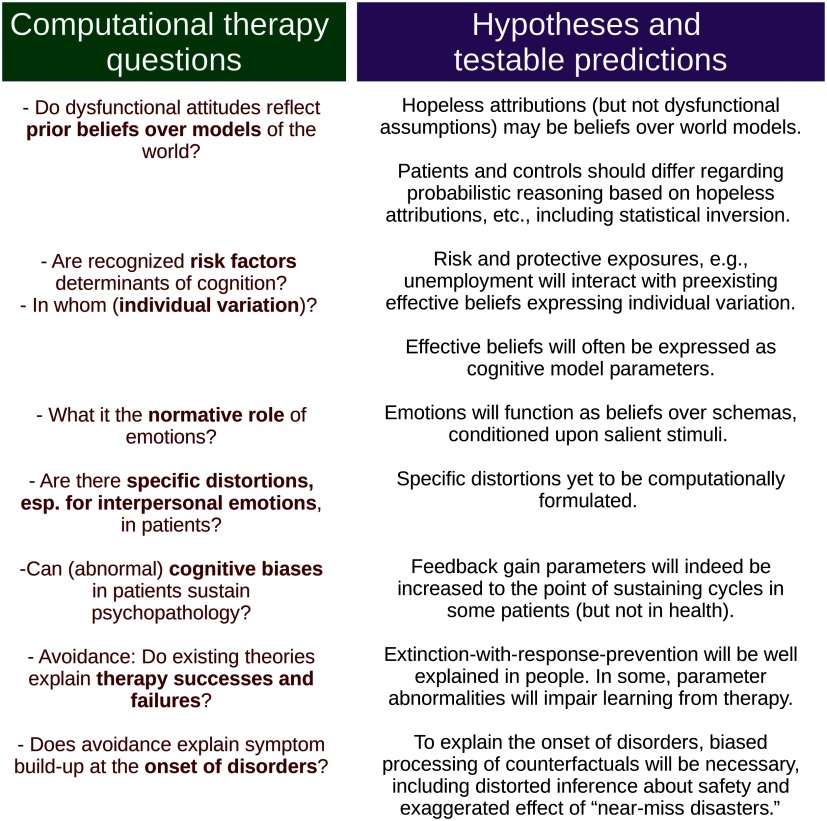
**Translating questions about therapy mechanisms in computational terms helps form testable predictions.** Left: important questions about the onset and maintenance of disorders. Right: predictions, in computational terms, that can be addressed in further research.

Findings about predisposing beliefs raise important questions as to their practical influence on reasoning. First, what is the nature and strength of the inferential mechanism expressed verbally as a global attribution bias? Can the modest predictive power of global attributions (Pearson et al., 2015) be improved by measuring them through computational tasks rather than questionnaires? Computational psychiatry is in an excellent position to develop tasks that assess both the strength and the nature of the overgeneralization involved. The apparent unreasonableness of “the cause will affect *all*areas of my life” is striking. Probabilistic tasks can determine how much such reported biases translate to decision making. It may be that such biases are “merely” expressive (expression, like poetry, serving higher order social roles). Alternatively, an expressed belief like “harmful causes have widespread effects” may reflect specific model-based reasoning or may be a verbalization of a model-free, habitual structure. Answers that appear to indicate an explicit reasoning model (model-based reasoning), elicited as attributional statements in questionnaires or in therapy, may in fact be verbalizations of strong habitual propensities. Precise, ecologically valid (Nord et al., 2017) tasks should also be devised to test under what quantitative conditions globalizing biases stabilize vicious inferential cycles (Figures 1 and 4). The development of such paradigms is important, because both biological and psychological theories postulate that abnormalities that cause no overt problems before the onset of a disorder do cause symptom escalation once an “illness threshold” is passed.

A further important question is how hopeless attributions are generated. Global attributions may be based on beliefs that negative events are due to pervasive personal causes, for example, “I am inadequate” (Figures 1 and 4). This is a cause that is always available to explain negative personal outcomes. Other patients may emphasize pervasive social structures, for example, attributing one’s misfortunes to sexism, the political system, one’s refugee status, and so on.^12^ The prototypical setting reminiscent of global attribution is learned helplessness (Huys, Guitart-Masip, & Dayan, 2014), where the value of exploration has been extinguished. Alternatively, the problem may be formulated as an accuracy–complexity trade-off (FitzGerald, Dolan, & Friston, 2014; Gershman & Niv, 2010). Here an oversimple model of the world, containing very few causes of events, has failed to accommodate new alternatives (schemas). Therefore inferring that a cause can cause substantial harm will naturally generalize to most domains.

## THERAPY FAILURES AND THE POSSIBLE ROLE OF OVERACCOMMODATION

Therapy can fail to help patients for a number of reasons, and here we will discuss how a specific computational process may help understanding and research into a particular class of failure. We first briefly overview categories of therapy failures to put this specific hypothesis in perspective. First, psychotherapy can fail because the patient or the provider has not given it an adequate trial. This problem has complex roots (Grant et al., 2012). Patients commonly avoid the therapy itself, disagree with the therapist’s formulation of the problem, or fail to carry out “homework” agreed upon with the therapist. Patients and therapists can have maladaptive beliefs about each other and the therapy itself, potentially undermining the patient–therapist relationship (Bevington, Fuggle, & Fonagy, 2015). Here a computational framework can help by offering an intuitive, validating, scientifically based model of deviant behavior to both patients and therapists (Moutoussis, Eldar, & Dolan, 2016) while focusing on needs, not pathologies (Moutoussis, Story, & Dolan, 2015). Second, therapies can have side effects and fail through causing harm. Here research is in its infancy (Crawford et al., 2016). Third, therapy may fail because it assumes neurocognitive resources that patients simply do not have. Fourth, we may have the wrong model of illness. Finally, the patient may adhere to the therapy and achieve change within it but learn too little about the world outside therapy. The first two failure modes (inadequate trial of therapy and side effects) are peripheral to the current work, while the third one (neurocognitive resources) needs in-depth treatment, which space does not permit. Most of the second and third sections of this article can be seen as addressing the fourth mode (models of illness). We now turn to the last mode, failure despite change.

Why do some patients fail to benefit despite acquiring ostensibly useful knowledge from therapy? Here we cannot claim that benign causes of experience are discounted altogether, as change has been effected in therapy. Computational psychiatry can explain such failures differently, by hypothesizing that such patients form overly complex models of their therapeutic experiences (they overaccommodate them). They are then prone to infer that different causes operate in the real-world setting.

**Figure 5.**
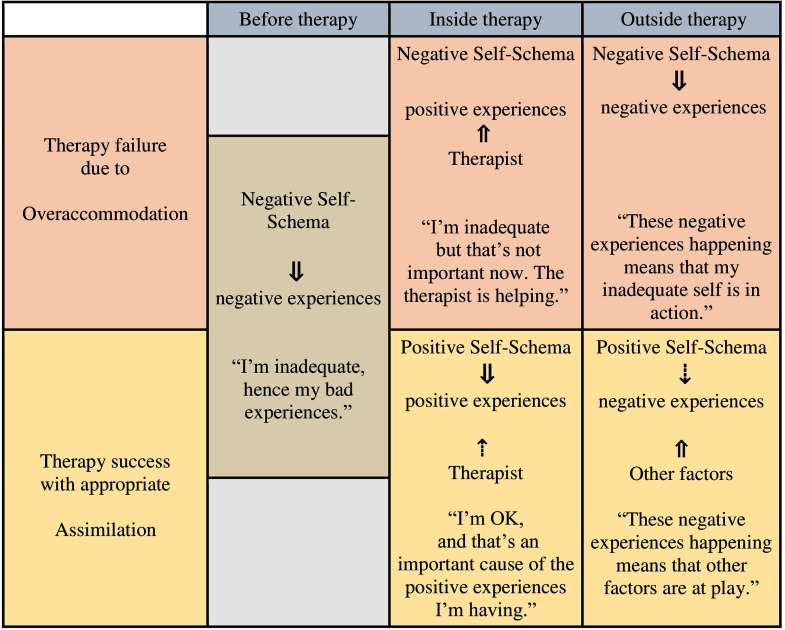
**Highly simplified examples of inference in therapy, either prone (top row) or resistant (bottom row) to failure owing to overaccommodation.** The arrows denote the strength of responsibility that the patient attributes to causes. In each row, before therapy, the patient attributes negative experiences to being inadequate. In the case of overaccommodation, positive experiences in therapy are mostly attributed to the therapist (equivalent to creating a new schema), without changing beliefs about the self. In assimilation (bottom row), the new cause (therapist) does not account for the new (success) experiences, so that beliefs about the self change (equivalent to adapting existing schema). The last column shows inference upon encountering new stressors outside therapy.

Learning continuously adjusts our models of the world. When new information is encountered, a learner’s default is to assimilate it into an existing schema (Piaget & Cook, 1952). A zebra may be classified (“assimilated”) as a kind of horse. If the concept of a horse now changes, so that it can have stripes, there is no formal error apart from the fact that “horse” is now a more complex schema (the genus *Equus*). If new information is sufficiently different to the schema in question (“disequilibrium”), a new component needs to be added to the model of the learner’s world (“accommodation”). We now explain why excessive creation of new schemas may reduce the impact of therapy in a patient’s everyday life (see Figure 5).

While behaviorists rejected schemas as unobservable, computational neuroscientists (Courville, 2006; Gershman, Norman, & Niv, 2015) have advanced an assimilation– disequilibrium–accommodation theory^13^ to explain why fear responses can often be reinstated following extinction. Here a well-conditioned stimulus that is suddenly no longer followed by aversive outcomes constitutes a qualitatively different experience for the learner, and so it is assigned to a new schema rather than being assimilated within, and thereby changing, the existing one. Niv and coworkers (e.g., Gershman & Niv, 2010) described this in terms of a learner invoking a different latent cause giving rise to the same stimulus. Applying this to therapy failure, we hypothesize that it is not enough for patients to learn that an anxiogenic situation does not give rise to the feared consequence. This may teach them that in the current context, a new, benign latent factor has been introduced rather than the original latent factor(s) now being less of a threat (Figure 5). This is what we term *overaccommodation*. It is post-Piagetian in the sense that it deals with the type of schemas discussed earlier, which may not be explicit goal-directed models.

How can we test whether overaccommodation leads to the aforementioned failures of therapy? The simplest way is to test the propensity of individual patients to attribute identical conditioned stimuli, only a fraction of which are punished, to two hidden causes rather than one. The latter should be less prone to fear reinstatement following extinction (Gershman & Hartley, 2015) and, we hypothesize, to future therapy failure. However, as in the operationalization of near-miss disasters, we suggest that experiments (and future clinical tests) can be optimized by investigating the invocation of therapy-relevant latent causes and also precipitants of reinstatement. During overaccommodation, patients may still attribute extratherapeutic events to the specific maladaptive causes mentioned earlier. These include beliefs about the self (“… because I’m inadequate”), various types of discrimination, and so on. Interview and questionnaire measures may provide preliminary data about overaccommodation itself, but first they should help select the most relevant stimuli and outcomes for immersive experiments (Moutoussis, El-Deredy, & Bentall, 2013).

Finally, it is important to link research efforts with how clinicians already attempt to address aspects of overaccommodation. Clinically, the most important factor that appears to induce reinstatement, and whose computational role needs investigating, as ways of remediating it are far from perfect, is nonspecific stress (cf. Figure 5, last column). In clinical settings, we have no clear reinforcement-timing differences flagging a need to infer the presence of new causes, as in animal paradigms. Instead, clinicians notice a lack of generalization over contexts such as therapy versus home environment. Clinicians attempt to address this through in situ practice of new learning, but this could be made a lot more systematic via the approach advocated here.

## DISCUSSION

Effective psychotherapies have great potential for cross-fertilization with computational psychiatry, as both grapple with the role of inference and learning in the genesis and maintenance of psychiatric distress. Using paradigmatic examples involving pessimistic inference and avoidance (sinking into depression when one is unable to find a job or avoiding coffee shops on the grounds of one’s short-of-perfect appearance), we first described likely computational mechanisms of belief maintenance, especially reduction of belief disconfirmation. To aid communication between therapy and research, we suggested formalizations that preserve the face validity of the relevant concepts from a therapy point of view. We then considered three hypotheses that bring formal computational approaches to bear upon important therapy questions (see also Figure 4). First, we hypothesized that for cognitive biases to grow or truly persist, schemas involving benign causes for events must be treated as irrelevant; that is, they must become unavailable for inference in the clinically relevant context, usually one of stress. Specifically, we hypothesized counterfactual gating of reinforcement to play an important role in augmenting dysfunctional beliefs. Such gating may also maintain dysfunctional beliefs by reducing the impact of new evidence. Second, we hypothesized that key cognitive vulnerabilities operate as prior beliefs, exemplified by “adverse circumstances are likely to affect most areas of my life.” We suggested testing these within models of consequential, for the patient, decision making. Finally, we considered therapy failures and especially the case of failure despite learning within therapy. We hypothesized this to reflect overaccommodation or, in computational terms, invocation by the patient of new causes explaining therapy experiences, causes unlikely to be inferred in the extratherapeutic real world.

With respect to the hypothesis of counterfactual gating, computational psychiatry should not look only for biased thinking but for biased *thinking under fire*, as argued in the section “Etiological Role of Avoidance.” In view of evidence that safety behaviors can distort inference and learning, we hypothesized more formally that for such biased learning to contribute to the *development* of a disorder, alternative schemas must be weakly available for inference in the clinical context. Under the counterfactual gating hypothesis, schemas alternative to the one driving avoidance are in principle available, but are not updated, as their effective priors in a stress scenario are suppressed, even down to zero. At the level of implementation, this could be because cognitive resources are devoted entirely to the management of defensive behaviors and none are left to process an updating of alternatives. A specific instance of this may be the case of near-miss disasters (inferred, for example, on interoceptive or vicarious grounds). These could block reinforcement of safety behavior–free policies, despite them furnishing safe outcomes. Even when avoidance is performed, perception of near-miss disaster may lead to an inference that even more vigorous defensive behaviors are needed for an adequate margin of safety. Near-miss theories have mostly been developed in gambling, pain, and public safety (Chase & Clark, 2010; Hölzl, Kleinböhl, & Huse, 2005; Tinsley, Dillon, & Cronin, 2012) but are still rare in psychiatric research. Another reason for counterfactual gating may be metacognitive, namely, that patients consider exploration of alternative, benign scenarios as conferring unacceptable risk of getting misled.

With respect to the hypothesis that cognitive vulnerabilities operate as prior beliefs over models of the world (section “Vulnerability Conferring Higher Order Beliefs”), computational psychiatry is in an excellent position to examine how powerful such priors-over-models are when it comes to real-world decision making. We exemplified this by the globalizing attribution bias “adverse circumstances are likely to affect most areas of my life.” Yet the simple idea of prior beliefs raises important corollaries. First, the postulated models of threat and opportunity hypothesized to guide patients need to be formalized in collaboration with therapy experts. This will ensure construct validity for concepts involved and avoid divergent appropriation of terminology.^14^ Research should prioritize priors about risk distribution, as severely negative outcomes are of central concern in most psychiatric disorders (including mania). Prior beliefs about the self need to be given prominence in different attributional biases. For example, the belief “I am inadequate” may underpin a globalizing attributional style. Priors may be about confidence in achieving outcomes (Friston et al., 2013) but also about preferences over self and of others (Moutoussis, Dolan, & Dayan, 2016; Moutoussis, Trujillo-Barreto, El-Deredy, Dolan, & Friston, 2013). Priors-over-models are useless without operationalizing said models for research. Therefore the likelihood functions that CBT and MBT imply that patients use in their generative models should be specified. These generative models of experience include the emotions of the agent (section “How Emotion Helps Construct Meaning”). Contrary to the irrationality usually ascribed to *ex consequentia* emotional reasoning, emotions must be considered as statistics containing information about schemas, informing useful beliefs about the state of the world and actions to take (Allen et al., 2008; Eldar, Rutledge, Dolan, & Niv, 2016; Giesen-Bloo et al., 2006; Joffily & Coricelli, 2013).

Yet if models of the world, including globalizing biases, are important for psychopathology, then people should be able to reason both about how causes produce events (generative mode) and also about what causes they should infer on the basis of events. The latter is statistical inversion, giving rise to conditional (upon events) beliefs. Can people perform these types of reasoning? Might patients have deficits in this domain? We do not know. Some parts of statistical inversion, especially the likelihood part, may be more implicit and well learned, whereas others, such as taking the base rate into account, may have to be done explicitly and be prone to errors. When it comes to emotions as causes, and to inferring intentionality, MBT has made a start (Allen et al., 2008), but there is much debate about how much emotion can affect thoughts. Some CBT instruments probe conditional beliefs as if they were mechanisms (“biases”). People are asked about their agreement with propositions like “if I fail at work, then I’m a failure as a person.” We challenge whether such negative self-beliefs are computational units. We suggest making the inversion involved explicit according to Bayes’s rule to help identify such units:

$$\begin{array}{ll} & \;\;\;\;\;\;p(\text{failed person}\;|\;\text{work failure})\\ & \;\;=p(\text{work failure}\;|\;\text{failed person})\;p(\text{failed person})\;/\;p(\text{work failure}). \end{array}$$

This indicates reasons why the conditional-belief-like scale scores mentioned previously (Pearson et al., 2015) were predictive of later depression but did not emerge as an independent risk factor. We can easily imagine that current low mood is highly correlated with the prior belief *p*(failed person) anyway. Such a participant would agree with the proposition above, but this would be fully mediated by his or her lower mood.

In addition, the power of globalizing attributions as well as other inferential factors should be tested in tasks*credibly evoking current concerns.*The tasks ideally should recruit the psychobiological systems ecologically engaged by these concerns, including evoking the affect and decision-making styles pertinent to the disorder. At the moment, neuroeconomic tasks used in computational psychiatry can only aspire to probe distant echoes of biases coactivated with distress. Ecologically valid tasks are technically and ethically challenging, requiring courage and trust from experimenter and subject alike. Some progress is being made using immersive paradigms (Nord et al., 2017), and computational psychiatry researchers need to develop them making full use of digital graphic communication, including virtual reality (Bouchard et al., 2017; Gega, 2017).

Overall, computational psychiatry can clarify which cognitive biases are in fact normative, given the patients’ concerns; which constitute risk factors peculiar to the individual; and which biases—normative or not—maintain disorders (section “Postulated Vicious Cycle of Cognitive Bias and Negative Mood”). Therapeutically, establishing that some cognitive mechanisms play an important role but are not distorted in patients is an opportunity for developing useful rehabilitation strategies, based on these intact capacities. However, it requires selection of important research questions, which can contribute to understanding pathophysiology if confirmed but, if not, can still indicate rehabilitation opportunities.

We classified therapy failures into five broad classes (section “Therapy Failures and the Possible Role of Overaccommodation”) and focused on failures to learn about the real world as opposed to the therapy context. Inspired by the work of Gershman and Niv (2010), we hypothesized that patients perceive critical differences between their experiences in therapy versus their own lives, which lead them to invoke new causes to explain the therapy experiences, causes unlikely to be inferred in their own lives.

When it comes to clinical practice, we have already informed our therapy practice by the computational perspective, although mechanistic improvements of therapy are still in the future. The computational perspective helps us to address patients’ questions, such as “is this psychological, or is there something wrong with my brain?” Practice has therefore already been helped at the level of psychoeducation, which is central to therapy (Moutoussis, Eldar et al., 2016).

Challenges remain, but for most of the issues we raise, different therapeutic alternatives are already practiced, so validating or refuting hypotheses would immediately constitute evidence for one strand of practice over another. With respect to challenges ahead, we would like to single out those posed by individual variation. The progression from a state of well-being to one of subclinical symptomatology to clinical disorder and back to health is likely to be highly dependent on individual brain differences, including engrams of individual risk- and protection-conferring environmental exposures. Risk engrams include not only conventional memories but also changes due to malnutrition, prolonged exposure to stress hormones, and so on. Isolated mechanisms like the examples we hypothesize about here are unlikely to be all-or-nothing causes. We suggest two additional strategies to meet this challenge. First, we suggest the systematic study of the dependence of therapy-relevant cognition on recognized risk and protective exposures (e.g., unemployment, known to precipitate depression). Second, we must exploit big data. *Computational psychotherapy* was recently put forward as the application of sophisticated computing methods to clinical psychotherapy data (Imel, Steyvers, & Atkins, 2015). Here we advocate using these methods to discover dimensions of individual variability relevant to learning and inference. Knowing these dimensions will greatly aid formulating mechanistic models of direct relevance to therapy.

In conclusion, the computational psychiatry of psychotherapy is a wonderfully fertile area for future study. Here we only scratch the surface and highlight some major research opportunities. It has been said of psychiatry that it should guard against being brainless (neglecting biology) and being mindless (neglecting psychotherapy). It is time for researchers in computational psychiatry to harness the skills of therapy researchers, and vice versa.

## Notes

1 Key psychotherapy terms are explained in Box 1.2 Key computational psychiatry terms, including *inference* and *learning*, in this article are explained in (Box 2).3 In honor of Ludwig Boltzmann, who died of suicide. His statistical work informs computational psychiatry.4 https://borderlinebabble.com/2015/07/30/teleological-thinking-mbt/5 Confusingly, CBT uses the same term as used for academic, scientifically rigorous behavioral experiments.6 In honor of—but unrelated to—Susie O’Neill, the Olympic medalist in swimming who overcame social anxiety. See http://www.verywell.com/what-is-susie-oneills-experience-with-social-anxiety-30242667 See Box 2 for explanations about effective beliefs, explicitly represented beliefs, and formal beliefs.8 See http://www.nice.org.uk/guidance/cg319 For an example, see http://www.bbc.co.uk/guides/z2vxp39#ztrpycw10 To invert Box’s saying, in psychiatry, most clinical models are useful, but some are misleadingly untrue. (Box, Hunter, & Hunter, 1978)11 Emotions predispose to their own active communication. We are not designed to play poker for good reason.12 People may of course correctly perceive that they have low IQ (“I am stupid”) or suffer discrimination. Therapy can be empowering but is not an adequate answer to such needs.13 Seemingly unaware of Piaget’s work. Piagetian *schemas* can be as simple as latent causes or as complex as mini-models of what to do in a situation. The qualitative theory of schemas has been refined since the time of Piaget, presenting further opportunities for computational psychiatry.14 We have tried both to give credit to the provenance of concepts we used and to avoid subtly appropriating them with shifted definitions. We trust the reader will be gracious with our resulting neologisms.

## AUTHOR CONTRIBUTIONS

Conceptualization: Michael Moutoussis, Nitzan Shahar, Tobias U. Hauser, Raymond J. Dolan; Formal analysis and software: Michael Moutoussis; Funding acquisition: Raymond J. Dolan; Methodology: Michael Moutoussis, Nitzan Shahar, Tobias U. Hauser; Project administration: Michael Moutoussis, Raymond J. Dolan; Supervision: Raymond J. Dolan; Visualization: Michael Moutoussis, Nitzan Shahar, Tobias U. Hauser; Writing: Original draft: Michael Moutoussis, Nitzan Shahar, Tobias U. Hauser; Writing: Review and editing: Michael Moutoussis, Nitzan Shahar, Tobias U. Hauser, Raymond J. Dolan.

## FUNDING INFORMATION

All authors were supported by the Neuroscience in Psychiatry Network and a strategic award by the Wellcome Trust to the University of Cambridge and University College London, of which RJD is a principal investigator (095844/Z/11/Z). This work was supported by the Wellcome Trust (RJD, Senior Investigator Award 098362/Z/12/Z). The Wellcome Trust Centre for Neuroimaging is supported by core funding from the Wellcome Trust (091593/Z/10/Z). The Max Planck UCL Centre for Computational Psychiatry and Ageing Research is funded jointly by the Max Planck Society and University College London. MM is also supported by the Biomedical Research Council. The authors have an affinity for computational psychiatry (MM, NS, TH, RD) and psychological therapy (MM, NS, TH) but otherwise declare absence of competing interests with respect to this work. NS is funded by a Rothchild postdoctoral fellowship.

## ACKNOWLEDGMENTS

We thank Glyn Lewis, Himanshu Tiagi, Liam Mason, Peter Dayan, and Laurence Hunt for stimulating discussions and Peter Fonagy for his advice and suggestions.
